# IDENTIFYING MARKERS OF NEURODEGENERATION FOR MOTORIC COGNITIVE RISK SYNDROME

**DOI:** 10.1093/geroni/igad104.2235

**Published:** 2023-12-21

**Authors:** Edward Ofori, Madeline Hooten, Marcus Ortega, Jordan Barajas, Dara James

**Affiliations:** Arizona State University, Tempe, Arizona, United States; Arizona State University, Phoenix, Arizona, United States; Arizona State University, Phoenix, Arizona, United States; Arizona State University, Phoenix, Arizona, United States; University of South Alabama, Mobile, Alabama, United States

## Abstract

Cognitive disorders, particularly dementia, are a major cause of disability in the elderly population. Gait disturbances, decreased grip strength and memory complaints are early signs of dementia that can occur well before the disease manifests. To better understand the close interactions between the physical and cognitive domains, the concept of Motoric Cognitive Risk Syndrome (MCRS) has been proposed. We had 25 older adults (10 cognitively unimpaired and 15 with MCRS) generate multiple force pulses at 15% of their maximum voluntary contraction with a fixed duration of 2 seconds for each pulse in which participants must rely on visual spatial abilities to gauge the amount force required in real-time to reach the criterion target. We also assessed neurofilament light chain levels also a blood-based marker of neuronal loss. Our results indicate the MCRS group demonstrated blood oxygenation level dependent (BOLD) hyperactivity in visual cortical/hippocampal regions, parahippocampal regions, and dorsolateral prefrontal cortex. The MCRS group also demonstrated BOLD hypoactivity in primarily involved with movement, such as cerebellar lobules, supplementary motor areas, middle frontal gyrus, and prefrontal cortex areas.be useful in tracking disease progression. We also found a significant strong positive correlation (0.83) between serum NFL levels and Right BOLD coefficients only for the CI group. This finding suggests that these motor task fMRI measures may be specific in tracking neurodegeneration in MCRS group. Overall, the current preliminary findings provide novel insights into the neurobiological mechanisms underlying early changes in Alzheimer’s disease and related dementias.

